# Supplementary material for: Direct Photochemical Synthesis of Substituted Benzo[b]fluorenes
Source: Org Lett. 2024 Nov 25;26(48):10364–8. doi: 10.1021/acs.orglett.4c03978 (PMC11629382; doi:10.1021/acs.orglett.4c03978)
Supplement: Supplementary file 2 — ol4c03978_si_002.zip [file ol4c03978_si_002.zip › FID for Publication/5l/20240526_RC-2-C-5-86B-F7_01/plots/RC-2-C-5-86B-F7_CARBON_20240526_01_plot01.pdf]

Sample Name:
RC-2-C-5-86B-F7
Data Collected on:
newucd400-vnmrs400
Archive directory:
/home/data/MBaumann/ucd400/RuairiCrawford/2024
Sample directory:
20240526_RC-2-C-5-86B-F7_01
FidFile: RC-2-C-5-86B-F7_CARBON_20240526_01

Pulse Sequence: CARBON (s2pul)
Solvent: cdcl3
Data collected on: May 26 2024

Temp. 25.0 C / 298.1 K
Sample #84, Operator: MBaumann

Relax. delay 1.000 sec
Pulse 45.0 degrees
Acq. time 1.000 sec
Width 25000.0 Hz
2000 repetitions
OBSERVE C13, 100.5494845 MHz
DECOUPLE H1, 399.8803230 MHz
Power 41 dB
continuously on
WALTZ-16 modulated
DATA PROCESSING
Line broadening 0.5 Hz
FT size 65536
Total time 1 hr, 10 min

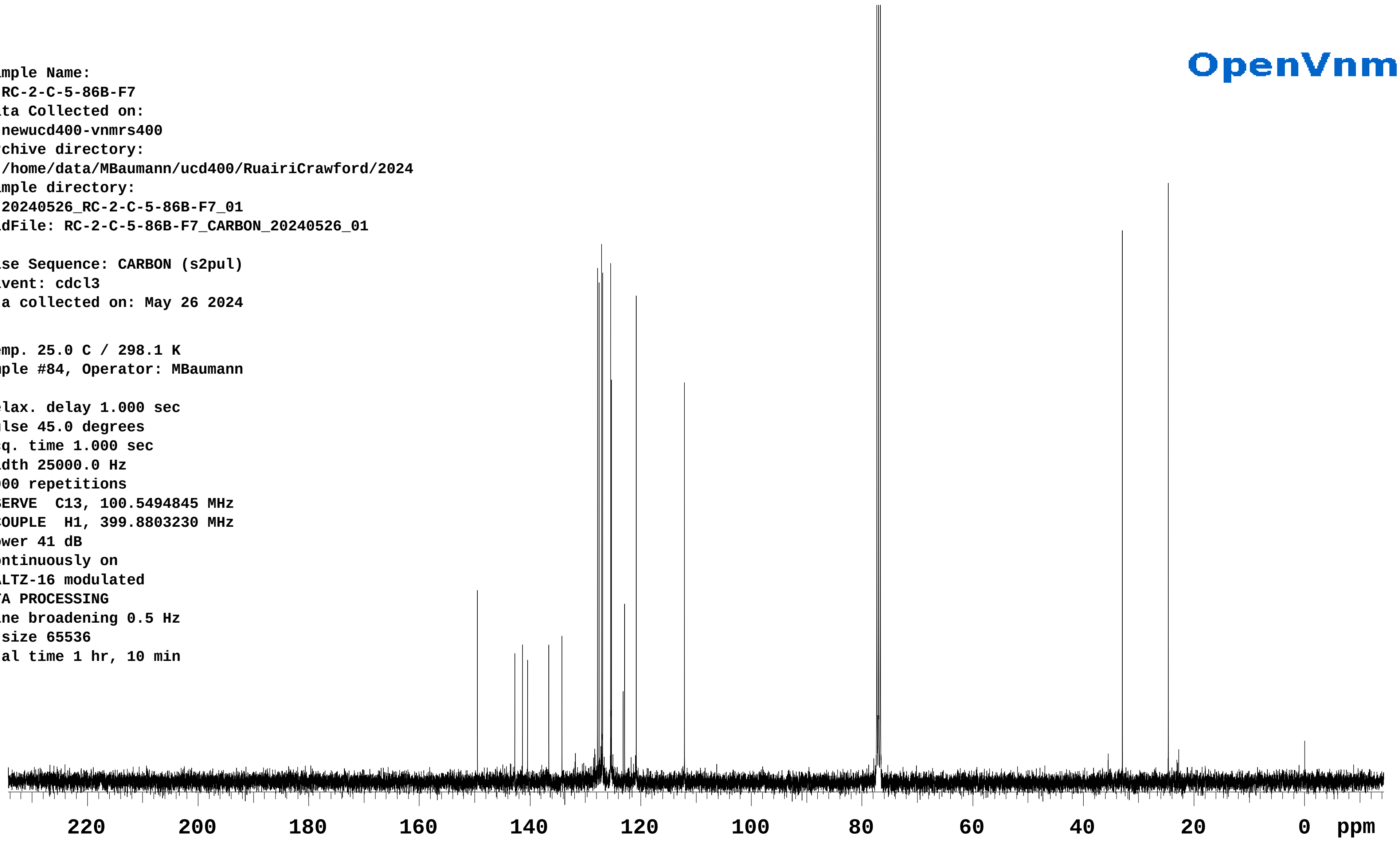

Plotname: RC-2-C-5-86B-F7_CARBON_20240526_01_plot01
